# A low-power approach to optical glucose sensing via polarisation switching

**DOI:** 10.1038/s41598-025-99367-0

**Published:** 2025-04-23

**Authors:** Ehsan Hassanpour, Mahsa Nasehi, Amir Meymandinezhad, Lilian Witthauer

**Affiliations:** 1 Department of Diabetes, Endocrinology, Nutritional Medicine and Metabolism, Inselspital, Bern University Hospital, University of Bern, Bern, Switzerland; 2 Diabetes Center Berne, Bern, Switzerland; 3 Graduate School for Cellular and Biomedical Sciences, University of Bern, Bern, Switzerland

**Keywords:** Polarisation switching, Glucose sensing, Optical biosensor, CGM, Polarimetry, Characterization and analytical techniques, Optical sensors

## Abstract

High-precision polarimetry is crucial for sensing and imaging applications, particularly for glucose monitoring within the physiological range of 50 to 400 mg/dl. Traditional approaches often rely on polarisation modulation using magneto-optic or liquid crystal modulators, which require high voltages or currents, limiting their practicality for wearable or implantable devices. In this work, we propose a polarisation-switching technique that alternates between two discrete polarisation states, offering a low-power alternative with miniaturisation potential. Using this method, we achieved a Mean Absolute Relative Difference of 7.7% and a Standard Error of Prediction of 9.6 mg/dl across the physiological glucose range, comparable to commercial continuous glucose monitors. Our approach demonstrates a limit of detection of approximately 40 mg/dl, with measurements performed in phosphate-buffered saline spiked with glucose. This work establishes polarisation switching as a viable alternative for glucose sensing, providing a foundation for future development of wearable and implantable glucose monitoring systems. By eliminating power-intensive components, our approach addresses key limitations of traditional polarimetric methods, paving the way for more accessible and energy-efficient diabetes management technologies.

## Introduction

Diabetes mellitus, which is characterized by the inability of the body to effectively regulate blood glucose (BG) levels, is one of the most rapidly growing health concerns worldwide. Over the past two decades alone, the prevalence of diabetes has surged by 255%, encompassing approximately 537 million individuals as of 2021^1,2^. This trajectory is predicted to persist, with projections from the International Diabetes Federation (IDF) indicating an increase to 643 million cases by 2030^1^. The implications of diabetes are profound, spanning a spectrum of health-related, societal, and economic consequences^3,4^. Among these, cardiovascular disease^5^, kidney dysfunction^6^, retinopathy^7^, and neuropathy^8^ are prominent long-term risks associated with the condition.

Diabetes is classified into several types, with type 1 and type 2 diabetes (T1D and T2D) being the most common forms. T1D involves the immune system attacking insulin-producing cells in the pancreas, leading to inadequate and eventually a complete lack of insulin production. T2D develops over time due to reduced cell responsiveness to insulin, often linked to diet, inactivity, and genetics^9^. People with T1D require lifelong insulin therapy. Traditionally, the insulin dosage has been guided by Self-Monitoring of Blood Glucose (SMBG), which involves measuring capillary blood samples with the help of glucose meters. Although being accurate, due to point measurements, SMBG’s ability to discover BG trends is limited, and short bouts of hyper- and hypoglycaemia tend to get missed. Therefore, in the past decade, continuous glucose monitoring (CGM) has become increasingly popular, mainly in people living with T1D. Current CGMs are most commonly based on an enzymatic reaction between glucose and glucose oxidase (GOx), which ultimately produces a current proportional to the glucose concentration^10,11^. CGM sensors have tremendously improved glucose management, especially in people with T1D^12–14^. Despite their transformative impact on diabetes care, current CGMs have critical limitations^15–17^, including inherent time delays and reduced accuracy in edge-case scenarios such as rapid glucose fluctuations. These constraints, compounded by slow insulin absorption, pose challenges to achieving fully automated insulin delivery systems^18–21^.

In light of these limitations, there is a growing need for novel glucose monitoring methods that offer high accuracy and minimal delay. Optical sensing techniques^22–30^ have emerged as a promising alternative, focusing on the physical properties of glucose molecules rather than their chemical interactions. A wide range of optical technologies has been explored for glucose monitoring, these methods include infrared spectroscopy^31–37^, Raman spectroscopy^31,32,38,39^, fluorescence spectroscopy^31,40–42^, optical coherence tomography (OCT)^31,32,35,43–46^, photoacoustic spectroscopy (PAS)^47,48^ and polarimetry^31,49–52^. The advantages and limitations of these techniques have been thoroughly reviewed in recent literature^22,23,31^. Among these approaches, polarimetry stands out for its exceptional sensitivity to glucose variations, even in the presence of confounding components such as those found in blood^50,51^.

Glucose molecules are chiral, i.e. their mirror image cannot be superimposed onto them. As a result, there are two stereoisomers of glucose in nature: D- and L-glucose, the latter of which is not naturally present in living organisms. The chirality of glucose molecules leads to a difference in the complex refractive index of the solution containing them when exposed to left- and right-handed circularly polarised light. The real part of this refractive index mismatch is referred to as circular birefringence and the imaginary part or the mismatch in the absorption is referred to as circular dichroism^53^. Linearly polarised light can be considered as a superposition of left- and right-handed circularly polarised light. Therefore, when passing through a medium containing chiral molecules, the polarisation vector of the linearly polarised light experiences a rotation. This rotation is proportional to the beam path length *l* and the concentration of the chiral molecules *c*^30^:

1 $$\begin{aligned} \phi = \alpha _R(\lambda , T) \cdot c \cdot l \end{aligned}$$

where $\alpha _R$ is the proportionality constant known as specific rotation of the chiral molecule, and is a function of wavelength, PH, and temperature. For a given wavelength $\lambda$, $\alpha _R$ can be estimated using Drude’s equation^52^:

2 $$\begin{aligned} {[}\alpha ]^T_{\lambda , pH} = \frac{A}{\lambda ^2 - \lambda _c^2} \end{aligned}$$

In this expression, the magnitude and sign of the optical rotation are determined by the constant $A$, while the absorption center wavelength $\lambda _c$ defines the characteristic spectral dependence of this rotation. For D-glucose solution, the specific rotation reaches an equilibrium value of about $\alpha _R=+52.9^\circ$ at $\lambda =630$ nm^54^.

Achieving the sub-millidegree sensitivity required for detecting glucose within the physiological range typically necessitates a polarisation modulator^55^. Magneto-optic modulators^55,56^, while highly precise, require high current (several amps), limiting their practicality for wearable or implantable devices. Liquid crystal modulators offer a low-power alternative but currently lack the resolution needed for accurate glucose measurement at physiological concentrations^57^.

To address these challenges, we propose polarisation switching, a technique that alternates the polarisation between two discrete values, equivalent to the modulation depth in sinusoidal modulation. While continuous polarisation modulation offers enhanced precision, our data indicate that polarisation switching provides adequate sensitivity for accurately measuring glucose concentrations within the physiological range (50-400 mg/dl). This method specifically uses the circular birefringence, i.e. the difference in the real part of the refractive index for left- and right-circularly polarised light caused by glucose’s optical activity, as the key physical property for sensing. Furthermore, it eliminates the need for power-intensive components, paving the way for compact, i.e. implantable or wearable, energy-efficient glucose monitoring systems.

## Results

**Fig. 1:**
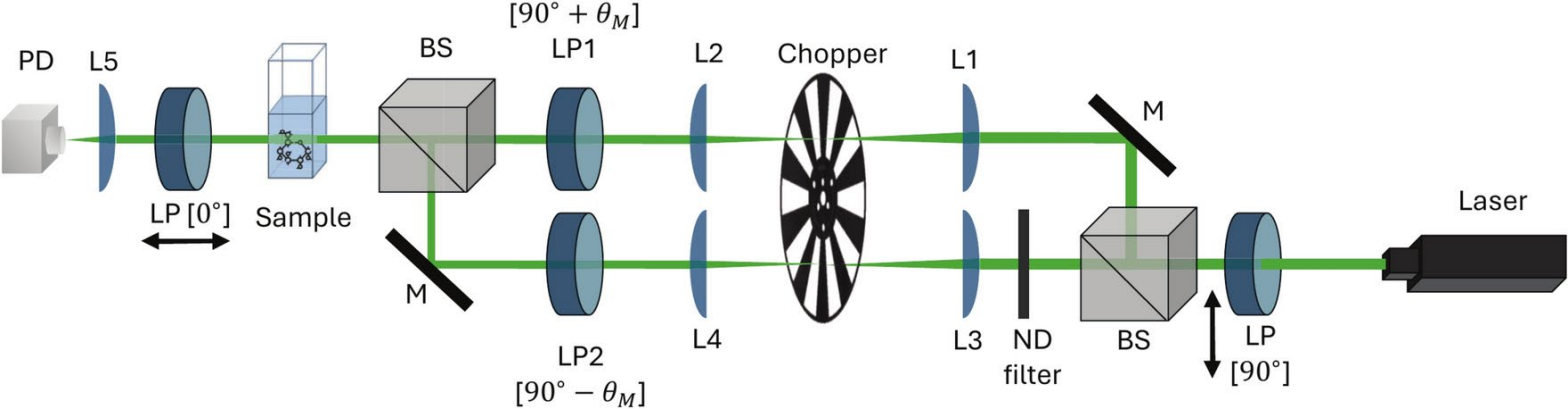
Schematics of the experimental setup. A green diode laser (532 nm) served as the light source, which is linearly polarised using a Glan-Thompson polariser (LP). The beam is split into two arms by a 50:50 non-polarising beamsplitter (BS). Polarisation modulation is achieved by linear polarisers set to $\pm \theta _M$ ($\theta _M<1^\circ$). The modulated beams are recombined using a second beamsplitter and pass through the sample cuvette. The transmitted light is analysed using another linear polariser (LP) and detected by a photodiode (PD). Additional components include mirrors (M), a neutral density filter (ND), and a lens (L) for alignment, intensity control, and focusing.

The experimental setup depicted in Fig. 1 employed polarisation switching with a 532 nm laser to measure glucose concentrations in phosphate-buffered saline (PBS) solutions. Although glucose exhibits stronger optical rotation at shorter wavelengths, as described by Drude’s equation, a wavelength of 532 nm was selected as a practical compromise to balance adequate sensitivity with reduced optical losses from water absorption. While our optical components support a broad spectral range (400–2700 nm), 532 nm was chosen for its favourable trade-off between signal strength, optical transparency in aqueous media, and component compatibility^50^. The optical chopper along with two linear polarisers alternated the polarisation states of light between 90$^\circ \pm \theta _M$ with $\theta _M<1^\circ$ and a frequency of 333 Hz, producing periodic intensity variations in the transmitted signal. These variations were analysed using Fourier transform methods to extract glucose concentration.

The transmitted intensity through the sample was modelled based on the square-wave modulation of the electric field, given as:

3 $$\begin{aligned} E(t) = \left[ \theta _{\textrm{M}} \cdot S(t) + \phi \right] \cdot E_0. \end{aligned}$$

Where $\theta _M$ is the modulation depth, *S*(*t*) is a square-wave function representing the abrupt polarisation switching and $\phi$ is the phase shift introduced by glucose. $E_0$ is the electric field amplitude of the light source. The square-wave function *S*(*t*) can be expressed using Fourier series:

4 $$\begin{aligned} S(t) = \frac{4}{\pi } \sum _{n=1,3,5,\ldots }^\infty \frac{1}{n} \sin \left( n \omega t\right) . \end{aligned}$$

which contains only odd harmonics of the modulation frequency $\omega$. By substituting *S*(*t*) into the electric field equation, the transmitted intensity *I*(*t*) is derived as:

5 $$\begin{aligned} \begin{aligned} I(t) \propto E^2(t) =&\left[ \theta _{\textrm{M}}^2 \cdot \frac{16}{\pi ^2} \left( \sum _{n=1,3,5,\ldots }^\infty \frac{1}{n}\sin {(n\omega t})\right) ^2 + \phi ^2 \right. \\&+\left. \theta _{\textrm{M}} \cdot \phi \cdot \frac{8}{\pi } \sum _{n=1,3,5,\ldots }^\infty \frac{1}{n} \sin {(n \omega t)} \right] \cdot E_0^2 \cdot T. \end{aligned} \end{aligned}$$

where *T* is the transmission coefficient of the sample. The glucose-induced phase shift $\phi$ manifests in the odd harmonics ($I(n\omega )$) and the direct current (DC) component ($I_{dc}$). Components with even harmonics of the modulation frequency $I(m\omega )$ appear as a result of expanding the squared-sum term in the intensity expression.

### Waveforms and Fourier transform

**Fig. 2:**
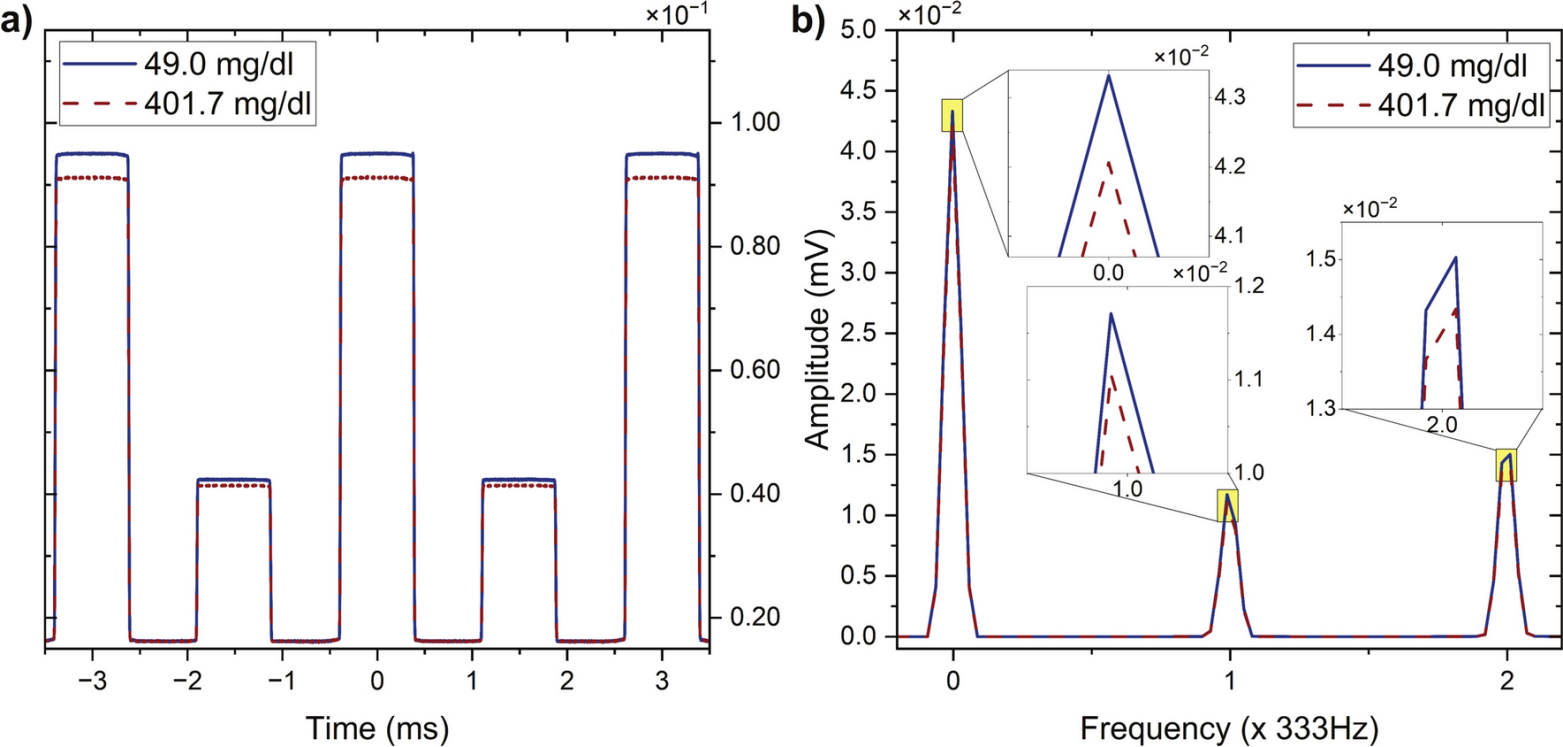
(**a**) Sample waveform recorded for 49.0 and 401.7 mg/dl glucose solutions. (**b**) Corresponding Fast Fourier Transform (FFT) plot. For clarity, only a selected section of the waveform and its corresponding FFT are displayed.

The transmitted intensity signals were recorded for glucose concentrations ranging from 49.0 to 401.7 mg/dl. Representative waveforms for glucose solutions at 49.0 and 401.7 mg/dl are shown in Fig. 2a, illustrating the periodic intensity variations introduced by polarisation switching. These variations arise from the glucose-induced phase shift $\phi$. As glucose concentration increases, the resulting phase shift alters the waveform characteristics, leading to measurable changes in signal modulation. The corresponding Fourier transform of the recorded waveforms is shown in Fig. 2b, providing a frequency-domain representation of the signal. By analysing the spectral components at various harmonics of the modulation frequency, the influence of glucose on the transmitted intensity can be examined.

Odd harmonic components ($I(n\omega )$), with $n=1,3,5,...$) were observed even in the absence of glucose ($c=0$, $\phi =0$), which was not expected based on Eq. (5). This phenomenon was attributed to an imbalance between the light intensities of the two arms (Supplementary Fig. S1 and Supplementary Table S1), as well as birefringence in the quartz cuvette (Supplementary Figs. S2 and S3), which induces distortions in the polarisation of light.

### Glucose concentration extracted from odd harmonics

To analyse the relationship between harmonic amplitudes and glucose concentration, the step function *S*(*t*) was approximated by a sum of sinusoidal waves of odd harmonics of the modulation frequency $\omega$ up to the *N*-th order. This expansion introduces even harmonics ($I(m\omega )$) and a DC component, with $I(m\omega )$ containing all even harmonics up to $m=2N$. For example, for the first three terms in the series ($N=5$), the expansion leads to:

6 $$\begin{aligned} \begin{aligned} \left( \sum _{n=1,3,5,\ldots }^\infty \frac{1}{n} \sin (n \omega t)\right) ^2&= \left( \sin {(\omega t)}+\frac{1}{3}\sin {(3\omega t)}+\frac{1}{5}\sin {(5\omega t)}+ \cdots \right) ^2 \\&= \frac{1}{450} \biggl (259 - 45 \cos (2 \omega t) - 60 \cos (4 \omega t)\\&~ - 115 \cos (6 \omega t) - 30 \cos (8 \omega t) - 9\cos (10\omega t) \biggl ) + \cdots \end{aligned} \end{aligned}$$

where the trigonometric identities $2\sin {a}\sin {b}=\cos {(a-b)-\cos {(a+b)}}$ and $\sin ^2{a}=(1-\cos {2a})/2$ were used for simplification. The magnitude of the DC component depends on the number of odd $\omega$ harmonics being considered. The detected intensity is thus as follows:

7 $$\begin{aligned} \begin{aligned} I(t) \simeq&\left[ \theta _{\textrm{M}}^2 \cdot \frac{8}{\pi ^2} \left( \frac{259}{450} + \cdots \right) + \phi ^2 \right. \\&+ \theta _{\textrm{M}} \cdot \phi \cdot \frac{8}{\pi } \left( \sin (\omega t) + \frac{1}{3}\sin (3\omega t) + \frac{1}{5}\sin (5\omega t) + \cdots \right) \\&- \theta _{\textrm{M}}^2 \cdot \frac{16}{\pi ^2} \biggl (\frac{1}{450} \biggl (45 \cos (2 \omega t) + 60 \cos (4 \omega t) + 115 \cos (6 \omega t)\\&+ 30 \cos (8 \omega t) + 9\cos (10\omega t)\biggl ) +\cdots \biggl ] \cdot E_0^2 \cdot T \end{aligned} \end{aligned}$$

This equation demonstrates the presence of both odd and even harmonics, along with the DC component. Depending on the experimental conditions, higher-order harmonics may be required to more accurately approximate the step function and achieve the desired sensitivity.

According to Eq. (7), glucose concentration $c_G$ can be directly deduced from its linear relationship with the sum of amplitudes of the odd harmonics $\sum I(n\omega )$:

8 $$\begin{aligned} \sum I(n\omega ) = a \cdot c_G + b \end{aligned}$$

where *a* and *b* are fitting parameters. To obtain these amplitudes at each frequency component, i.e., the intensity of the corresponding harmonic $I(n\omega )$, each peak in the Fourier spectrum was fitted with a Gaussian function. The extracted amplitudes were then summed, plotted against the glucose concentration, and subsequently fitted with a linear function. This sum corresponds to the expansion of the square-wave function *S*(*t*) in Eq. (4), and accordingly the sums in Eq. (5) up to the *N*-th order.

The coefficient of determination $R^2$ of the linear fit is shown in Supplementary Fig. S4 for different order of approximations *N*. The highest $R^2$ was observed for $N=13$. Although incorporating higher-order harmonics is theoretically beneficial for approximating the step function in Eq. (4), this approach is not practically advantageous. As *N* increases, errors in the calculated harmonic intensities accumulate, leading to greater overall uncertainty. This results in either negligible improvements or even a deterioration in linearity beyond the optimal *N* (see Supplementary Fig. S4).

**Fig. 3:**
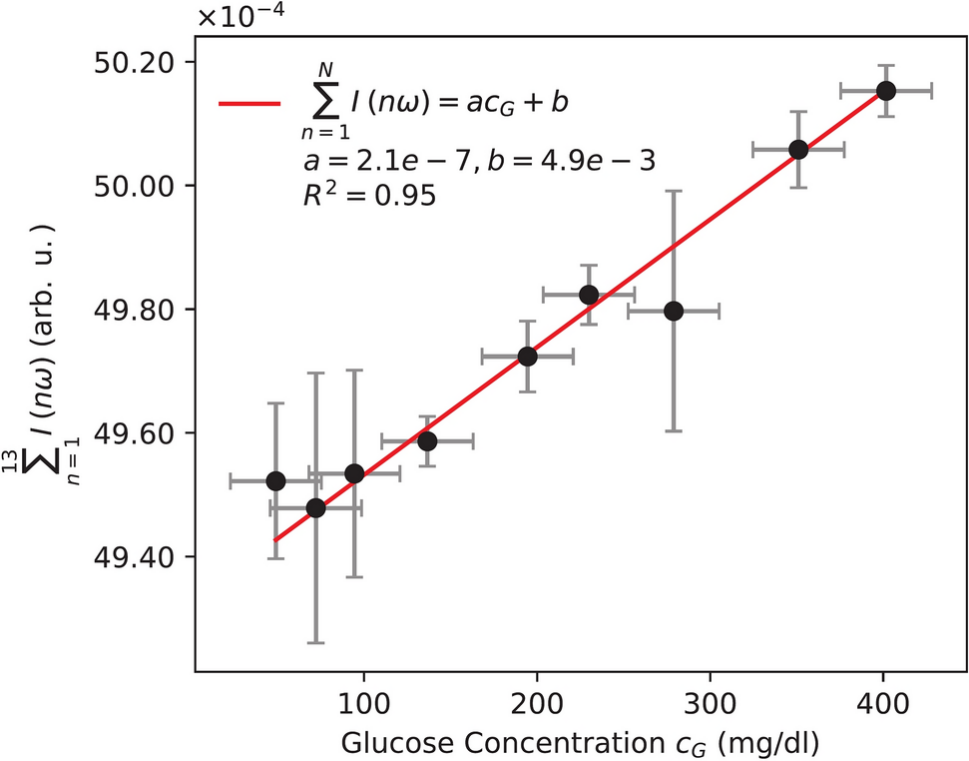
Glucose dependence extracted from the sum of odd harmonic intensities up to $N=13$. The linear fit achieves an $R^2$ of 0.95 with an SEP of 26.37 mg/dl (x-error). The error bars in the y-axis represent the standard deviation of 15 independent measurements.

Figure 3 shows the sum of the intensities of odd harmonics up to the $N=13$. Each data point represents the average of 15 independent measurements, with the y-error bars indicating the corresponding standard deviation. The Standard Error of Prediction (SEP) for glucose concentration was computed from a linear regression model relating the summed harmonic intensities to glucose concentration as shown in Eq. (8). The SEP is defined as the root mean square error (RMSE) of the residuals between the measured and predicted values in the intensity domain, divided by the slope of the calibration curve. That is,

9 $$\begin{aligned} \text {SEP}_{c_G} = \frac{\sqrt{\frac{1}{n-k}\sum _{i=1}^{n}\left( y_i - f(x_i)\right) ^2}}{\left| \frac{dy}{dx} \right| }, \end{aligned}$$

where $y$ represents the measured summed harmonic intensity and $x$ denotes the corresponding glucose concentration. The derivative $\frac{dy}{dx}$ quantifies the sensitivity of the calibration curve. The resulting SEP of 26.37 mg/dl suggests that $\sum I(n\omega )$ alone is not a reliable parameter to measure glucose concentration which was also mentioned by Stark *et al.*^52^.

Although $\sum I(n\omega )$ exhibits a linear relationship with $c_G$, its dependence on light intensity fluctuations and medium transmission, as described in equation (5), introduces significant variability. An approach to mitigate such dependencies would be to normalize the signal using the ratio of odd to even frequency contributions, $\sum I(n \omega )/\sum I(m\omega )$^52^. However, this method did not yield satisfactory results in our experiments. Unlike continuous polarisation modulation, which primarily involves only $\omega$ and $2\omega$, this normalization approach resulted in the inclusion of multiple odd and even harmonics, deviating significantly from the expected behaviour. The linearity of this ratio with respect to glucose concentration is further analysed in Supplementary Materials Section S4. To address these limitations, the next section explores an alternative approach utilizing the ratio of *I*(*dc*) and $I(n\omega )$, providing improved robustness against intensity fluctuations and transmission variations.

### Glucose concentration extracted from the ratio of DC component and odd Harmonics

Both *I*(*dc*) and $I(n\omega )$ depend on the input light intensity $I_0=E_0^2$ as well as the transmission of the medium *T* according to Eq. (5). By taking the ratio of these two components, dependencies on fluctuations in light intensity and absorption effects within the medium are effectively eliminated. Unlike odd harmonics, which are linearly dependent on glucose-induced phase shift $\phi$, $I_{dc}$ follows a non-linear dependence, see Eq. (10). Consequently, the ratio of these terms leads to the following relationship for the glucose concentration $c_G$:

10 $$\begin{aligned} I(\text {dc})/ \Sigma I(n\omega ) = d \cdot c_G + e / c_G + f \end{aligned}$$

where *d*, *e* and *f* are fitting parameters.

**Fig. 4:**
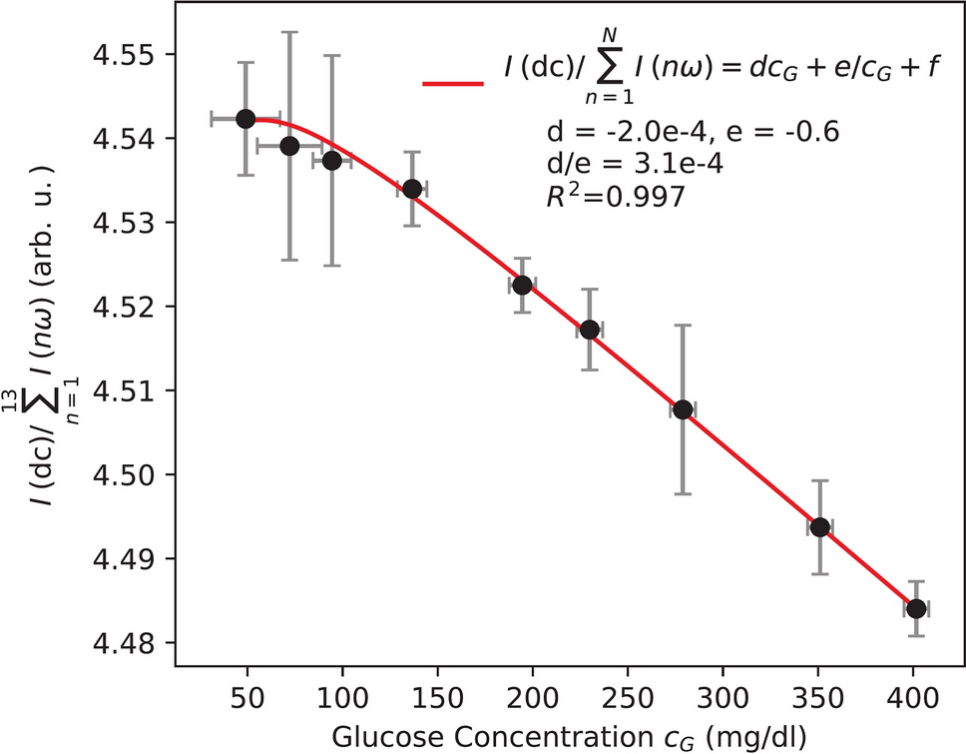
Ratio of the DC component of the measured signal to the sum of odd harmonics as a function of glucose concentration. Each data point represents the average of 15 independent measurements, with y-errors indicating the standard deviation. The data (black dots) is fitted using the non-linear equation, achieving a fit quality of 0.997.

Figure 4 shows the ratio of *I*(*dc*) and $I(n\omega )$ for glucose concentrations ranging from 49 to 401 mg/dl. As before, each data point represents the average of 15 independent measurements, with y-errors indicating the corresponding standard deviation. The SEP for this method varies between 18.1 mg/dl and 6.5 mg/dl across the full concentration range, resulting in an average SEP of 9.6 mg/dl across the whole physiological range. The SEP was determined using non-linear regression models (Eq. 10) that relate detector readings to glucose concentration, with the RMSE normalized to the glucose levels. The limit of detection (LOD) for this method was calculated as 40.4 mg/dl, based on the standard deviation of the signal at low glucose concentrations and the sensitivity of the calibration curve.

Further analysis of measurement variability (Supplementary Fig. S5) indicates that laser fluctuations and temperature-induced changes in optical elements are the primary sources of standard deviation. This is supported by intensity fluctuations observed even in the absence of a cuvette, whereas the highest variability (average standard deviation of 0.14%) was recorded with a PBS-filled cuvette, implying additional contributions from cuvette birefringence and temperature variations in PBS. Moreover, simulations of polarisation rotation changes for glucose over $15-35\,^{\circ }\hbox {C}$ (Supplementary Fig.  S6) showed negligible effects, confirming that the variability mainly originates from the laser and optical components.

## Discussion

In this work, we have shown that polarisation switching offers an effective method for measuring glucose concentrations within the physiological range. Unlike traditional methods that rely on continuous polarisation modulation, often necessitating devices that operate at high voltages or currents, polarisation switching eliminates the need for such demanding specifications. This advancement opens up new possibilities for the development of wearable or implantable glucose monitoring devices, marking a significant step toward the development of new diabetes management technology.

The average observed SEP of 9.6 mg/dl across the physiological glucose concentration range of 50 to 400 mg/dl supports the feasibility of this method for clinical glucose monitoring in humans. To further assess the accuracy of this approach, we evaluated the Mean Absolute Relative Difference (MARD), a commonly used metric for glucose sensor performance. MARD quantifies the average relative error between measured and reference glucose concentrations and serves as a key benchmark for evaluating glucose monitoring technologies. Commercial continuous glucose monitors (CGMs) achieve MARD values of approximately 8-10% in vivo, ensuring clinically acceptable accuracy for diabetes management^58^. Our method demonstrates a comparable average MARD of 7.7%, with notably higher MARD values at lower glucose concentrations (see Supplementary Information Table S2), a trend also observed in commercial CGMs. This performance is comparable to other polarimetric glucose sensing methods reported in the literature. A study employing a single-polarisation rotator system with a machine learning-based glucose prediction model achieved a MARD of 6.8%, showing slightly lower errors, though it relied on a Mueller matrix-based system, which increases system complexity^59^. Similarly, a Faraday rotator-based approach for glucose detection in turbid media reported an exceptionally low SEP of 1.17 mg/dl at 528 nm^52^. The same group demonstrated a broadband approach (380-680 nm), achieving an SEP of 1.7 mg/dl in pure glucose solutions and 16 mg/dl in protein-containing media^50^. In contrast, our polarisation-switching design eliminates the need for Faraday rotators, providing a simpler setup while maintaining competitive accuracy, even with a 1 cm cuvette, compared to the 5 cm cuvettes used in previous studies. It is also important to note that other polarimetric studies employed measurement step sizes of 100 mg/dl, which limits the precision of their detection capabilities and makes it challenging to estimate the true LOD. In contrast, our study employed finer measurement increments, starting at 49 mg/dl, allowing us to calculate a meaningful LOD of 40.4 mg/dl.

Our results were obtained in aqueous solutions in vitro, where scattering and absorption effects are minimal. While polarisation-based glucose sensing has shown promising accuracy under these conditions, translating this approach to highly scattering media, such as biological tissues, presents additional challenges. The presence of multiple scattering events and inhomogeneities may introduce signal distortions that require advanced correction techniques to maintain sensitivity and accuracy. Addressing these factors through optimised optical configurations and signal processing will be critical for future applications in biological environments.

Although our method shows strong potential, the current experimental setup includes sources of error that are not inherent to the method itself and can be either eliminated or substantially reduced in future implementations. For instance, by utilising a miniaturised system with waveguides, a more precise splitting and recombination ratio, approaching the ideal 50:50, can be achieved. This enhancement would improve the system’s sensitivity to minor polarisation changes induced by the presence of glucose. Furthermore, the measurement was adversely affected by the birefringence of the cuvette wall, a limitation that will not be present in the final device. However, while waveguides can reduce the influence of certain error sources, they are subject to birefringence and dispersion effects that can introduce additional wavelength-dependent noise. Therefore, careful polarisation management, selection of materials and waveguide geometries and the implementation of compensation mechanisms for temperature and motion artefacts will be necessary in such systems. In the long term, these waveguide-based systems could be extended into full photonic integrated circuits (PICs), combining modulation, polarisation control, and detection into a compact and scalable platform.

Subsequent developments of our experimental setup will focus on employing custom-designed cuvettes equipped with birefringence-free windows. This modification aims to fine-tune the modulation depth, $\theta _{\text {M}}$ to align closely with $\phi$ across the targeted concentration range, significantly improving the system’s sensitivity to variations in $\phi$. Such fine-tuning is particularly important when employing a non-linear function for glucose prediction. As demonstrated in the Supplementary Fig. S7 careful adjustment of $\theta _{\text {M}}$ can enhance sensitivity, particularly for lower glucose concentrations.

Furthermore, we plan to replace the mechanical chopper with direct intensity modulation of the light source, which will not only eliminate errors from mechanical vibrations but also allow for an increase in modulation frequency into the MHz range, enabling faster and more efficient data collection. To further differentiate glucose from other optically active biomolecules present in complex media such as blood, particularly serum albumin, we propose replacing the laser diode with a broadband light source. Importantly, common glucose analogues such as fructose exhibit opposite optical rotation^60^ and are present at much lower concentrations in blood^61^, while disaccharides like lactose are typically absent from circulation under healthy physiological conditions^62^. This will enable optical rotatory dispersion (ORD) measurements across a broader spectral range, improving both selectivity and accuracy. Future studies will also include simulations to evaluate the impact of scattering in tissue and experimental measurements in tissue models to further assess the feasibility of implementing this method in biological environments.

## Methods

### Experimental setup

The polarimetry setup is illustrated in Fig. 1. A green diode laser (CPS532, Thorlabs GmbH, Germany) with a centre wavelength of 532 nm and nominal power of 4.5 mW was used as the light source. The output of the laser was linearly polarised at an angle of $90^{\circ }$ (*s*-polarisation) by passing through a Glan-Thompson polariser (PGT 3.08 B.Halle Nachfl. GmbH, Germany) with an extinction ratio of 1:100,000. The vertically polarised beam then entered a non-polarising 50:50 beamsplitter (BS010, Thorlabs GmbH, Germany) and was split into two arms. At each arm a demagnifying telescope was used to reduce the beam size by half. Two Glan-Thompson polarisers with extinction ratios of 1:1000,000 (PGT 2.08 B.Halle Nachfl. GmbH, Germany) set to $\pm \theta _{\textrm{M}}$ with $\theta _{\textrm{M}}<1^{\circ }$ were placed in each of the arms. Subsequently, the two beams were recombined using a second non-polarising beamsplitter. The combined beam was guided through a cuvette holder where glucose solutions of various concentrations were inserted. The output beam from the sample was then analysed using another Glan–Thompson polariser set at $0^{\circ }$ (*p*-polarisation) and detected using a photodiode (PDA36A2, Thorlabs GmbH, Germany).

Polarisation switching was accomplished by alternating between the two arms using temporal delay control. This involved employing an optical chopper (MC1F10A, Thorlabs GmbH, Germany) to selectively modulate the beam from each arm, allowing only one arm’s beam to reach the detector at any given time. As a result, the light that impinged on the sample had a polarisation alternating between $+\theta _{\textrm{M}}$ and $-\theta _{\textrm{M}}$. The chopper was placed at the focal point of the telescope such that the time to chop the beam was minimal. Therefore, on the detector, the intensity changed abruptly, which was translated to an abrupt change in polarisation. For significant beam sizes at the chopper position, the blade cuts the beam gradually, resulting in a smooth change of intensity on the detector (see Supplementary Fig. Section S8). However, this smooth intensity change does not correspond to a gradual change of polarisation, unlike in the case of continuous polarisation modulators.

### Preparation of glucose solutions

A glucose stock solution was prepared in a volumetric flask by dissolving 1 g of D-(+)-glucose powder (CAS 50-99-7, molekula group) in 25 ml PBS to reach a final concentration of *c* = 1275 mg/dl. The prepared stock solution was stored in the fridge at $4^{\circ }\hbox {C}$ for at least 24 h for the mutarotation of $\alpha$- and $\beta$-glucose to stabilize. In addition, a blank solution of PBS was prepared for the dilution procedure.

Before starting the measurement, both the stock and the blank solutions were thermalised at room temperature. Then 2 ml of the blank solution was transferred to a 10 mm path length quartz glass cuvette (CV10Q35FA, Thorlabs GmbH, Germany) using a 1000 $\mu \hbox {l}$ pipette. The stock solution (12.75 mg/dl) was subsequently added to the cuvette in multiples of 40 $\mu \hbox {l}$, resulting in final glucose concentrations of 49.03, 72.16, 94.44, 136.60, 194.49, 229.91, 278.90, 351.08, and 401.71 mg/dl. To ensure meaningful resolution within the physiological glucose range (approximately 70-140 mg/dl), we selected smaller concentration steps, while for the remaining range, a minimum difference of 50 mg/dl between points was maintained to cover the full dynamic range effectively. To ensure homogeneity following each addition, the solutions were vortexed at a constant speed of 8 rpm until any observable signs of inhomogeneity, such as light refraction, were no longer apparent. The selection of this mixing speed was based on empirical considerations to prevent the formation of air bubbles within the solution.

### Data acquisition

For the measurement, the cuvette was placed in the holder of the experimental setup (CVH100/M, Thorlabs GmbH, Germany) and the output of was recorded as a waveform using an oscilloscope (MDO34, Tektronix). To improve the signal-to-noise ratio, a 128 times averaging was performed on the waveforms. Taking into account a chopper frequency of 333 Hz, the record length was set to 400 ms for optimal sampling of the waveforms. Therefore, the data acquisition for each measurement lasted approximately 51 s. The optical response itself is effectively instantaneous, and the acquisition time reflects only the averaging protocol, not any inherent delay in the sensing process. The chopper frequency was carefully chosen to avoid coincidence with the harmonics of the power line (50 Hz) and was kept at a moderate level to prevent the introduction of vibrational noise caused by air turbulence. The duty-cycle of the chopper was set to 25% to guarantee specific time intervals where both beams were blocked simultaneously. This allowed for the distinction and separation of the contributions from each beam arm (corresponding to $+\theta _{\text {M}}$ and $-\theta _{\text {M}}$ polarisations). Employing a duty cycle of less than 50% facilitates the examination of the scenario when $\phi =0$, where no difference between the intensities of the two arms is anticipated. This results in a constant signal on the detector, i.e., a dc-like signal without any information on the polarisation modulation.

## Supplementary Information

Supplementary Information.

## Data Availability

The datasets generated during and analysed during the current study are available from the corresponding author on reasonable request.
