# Supplementary material for: A low-power approach to optical glucose sensing via polarisation switching
Source: Sci Rep. 2025 Apr 23;15:14200. doi: 10.1038/s41598-025-99367-0 (PMC12019186; doi:10.1038/s41598-025-99367-0)
Supplement: Supplementary file 1 — Supplementary Information. [file 41598_2025_99367_MOESM1_ESM.pdf]

Supplementary Information: A low-power approach to optical glucose sensing via polarisation switching

Ehsan Hassanpour[1,2+], Mahsa Nasehi[1,2,3+], Amir Meymandinezhad[1,2], and Lilian Witthauer[1,2*]

[1]Department of Diabetes, Endocrinology, Nutritional Medicine and Metabolism, Inselspital, Bern University Hospital, University of Bern, Bern, Switzerland
[2]Diabetes Center Berne, Switzerland
[3]Graduate School for Cellular and Biomedical Sciences, University of Bern, Switzerland
[*]lilian.witthauer@unibe.ch
[+]these authors contributed equally to this work

S1 Beamsplitting imbalance

Although the beamsplitter and combiner were used symmetrically for the signal line, the intensities of the two arms were not equal. To measure this imbalance, each of the polarisers in the two arms was set to 0° (parallel to the optical table surface normal) while the analyser was set to 90° (perpendicular to the optical table surface normal). The measured values for the intensities of the two arms were 19.7 mV and 8.67 mV for the left and right arms, respectively. A neutral-density (ND) filter wheel in the left arm was used to adjust the attenuation in order to balance the two arms. Figure S1 presents a sample waveform recorded after balancing, while Table S1 reports the transmission and reflection coefficients for s- and p-polarized light at 532 nm for the BS010 beamsplitter (Thorlabs[1]).

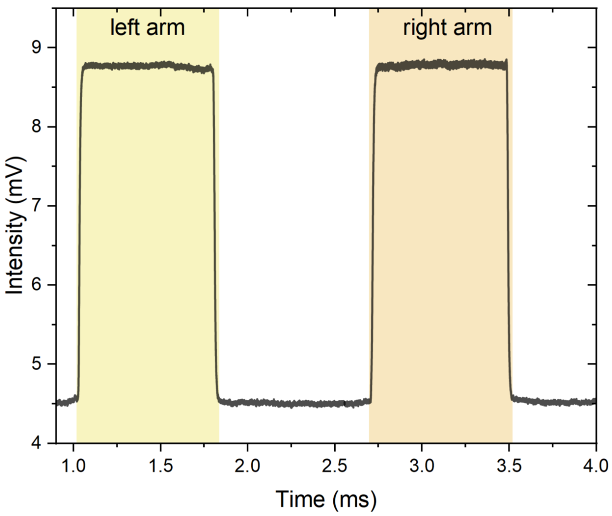

Figure S1. Sample waveform recorded for signal line without the cuvette in the setup and with both polarisers set to 90° and after inserting an ND filter in the left arm.

	p-polarised	*s*-polarised
Transmission (%)	56.34	51.28
Reflection (%)	39.73	45.65

Table S1. Transmissions and reflections for *s*- and *p*-polarized light at 532 nm for BS010 from Thorlabs[1]

S2 Birefringence of the cuvette walls

The birefringence of the cuvette exerts adverse effects on the measurements. First, it disturbs the linearity of the polarisation entering and exiting the glucose solution and thus reducing the signal-to-noise ratio of the system. Second, it introduces an alteration in the orientation of the input polarisation, manifesting as an error $\delta\theta$ in the modulation depth θ_M. Depending on the orientation of the optic axes of the cuvette wall, $\delta\theta$ is different for θ_M and $-\theta_\mathrm{M}$ beams. For instance, consider the scenario where one of the fast- or slow axes of the cuvette wall aligns parallel to the θ_M polarisation direction. In this case, the polarisation of the θ_M beam remains unaffected while the polarisation of the $-\theta_\mathrm{M}$ beam undergoes a rotation. Therefore, even in the absence of polarisation rotation due to the glucose sample, the two beams exhibit different intensities after passing through the analyser. Consequently, achieving θ_M matching ϕ becomes experimentally challenging. According to equation (6) in the main document, θ_M matching ϕ increases the sensitivity of the signal to minute changes of ϕ[2].

Another important distortion arising from the birefringence of the cuvette is associated with mechanical perturbations of the setup and the cuvette itself. Any misalignment between the beam and the cuvette can result in a shift in the polarisation of the beam entering the glucose solution, introducing potential additional errors. It should be noted that this issue is viewed as extrinsic to the chosen methodology, given that the utilization of cuvettes serves a purely demonstrative purpose. To prove that the cuvette walls are not perfectly isotropic (glass) but has some residual birefringence, a simple experiment was performed. A cuvette with with bare PBS solution (no glucose) was mounted on a rotation stage allowing rotation in clockwise (cw) and counter-clockwise (ccw) direction with respect to the beam. Figure S2 a) shows a sample waveform for the cases without any cuvette in the setup, and with the cuvette at 0° (vertical) as well as 2° and 4° cw and ccw rotation. The exaggerated cases of 45° cw and ccw are also shown in comparison to 0° and no cuvette in Fig. S2 b).
It is worth mentioning that the two beams were focused on the detector with a $3.6 \times 3.6\,\mathrm{mm}^2$ active area. The alignment of the beams on the detector was carefully verified in order to avoid misalignment-induced intensity changes while rotating the cuvette.

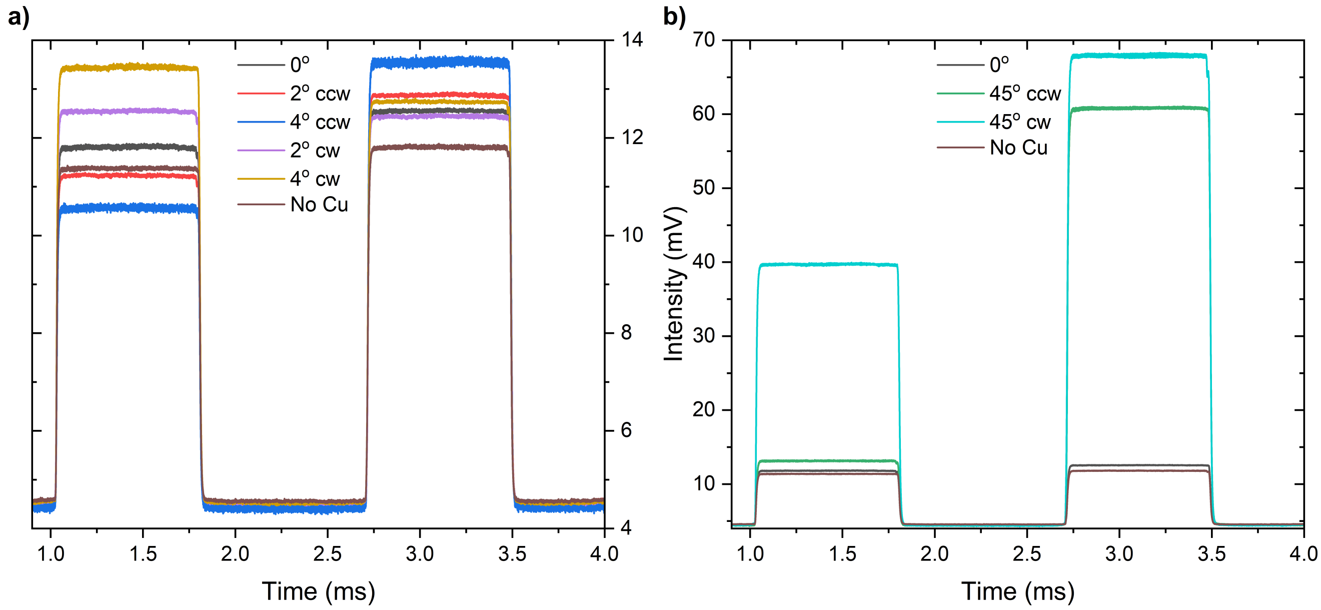

Figure S2. Sample waveform recorded for signal line for various tilt angles and directions of the cuvette containing the bare PBS solution. The polarisers in the left and right arms were set to about 0.5° ccw and cw ($|\theta_\mathrm{M}| \approx 0.5°$), respectively. Similar to Figs. S1 and S8, the left and right peaks in the plots represent the beams in the left and right arms in the setup, respectively.

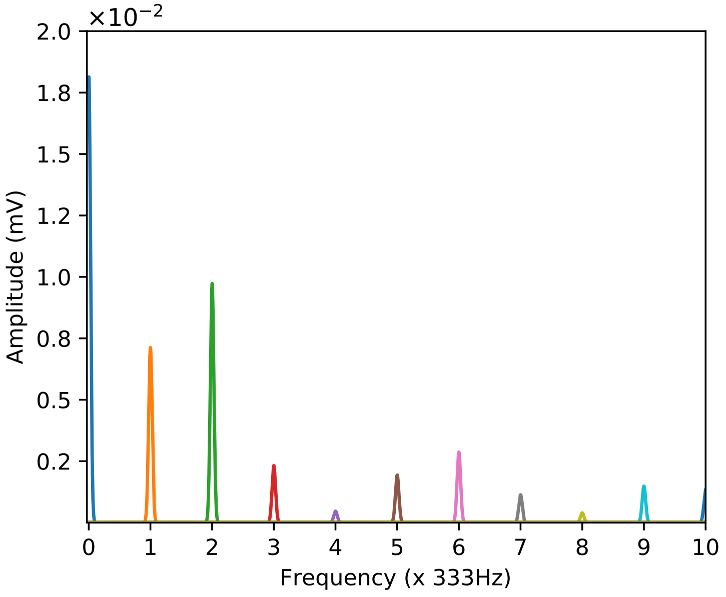

Figure S3. Fast Fourier Transform (FFT) plot of a signal recorded from a PBS solution without glucose. For clarity, only a selected section of the waveform and its corresponding FFT are displayed.

S3 R^2 and SEP calculations

Figure S4 shows the resulting R^2 and average SEP for a) $\sum_n^N I(n\omega)$ and b) $I_{dc}/\sum_n^N I(n\omega)$ up to $N = 17$. The coefficient of determination R^2 increases significantly up to the 9th order for both Figures S4 a) and b). The same behaviour is observed for the average SEP. Beyond these orders, the R^2 value stabilizes, showing little variation with the inclusion of additional harmonics while average SEP monotonically improves further up to the 15th harmonics. Note that, although considering higher-order harmonics is theoretically advantageous for approximating the step-function in equation(6) of the main text, this is not practically beneficial. The accumulation of errors in calculated intensities for different harmonics becomes more pronounced with higher-order approximations, leading to increased accumulated error. Consequently, one often observes either negligible improvement or even a deterioration of the fit quality after an optimal N. For $I_{DC}/\sum_n^N I(n\omega)$, changes in SEP for up to the 17th harmonic result in an approximately 80% reduction in SEP, indicating a significant improvement in predictive accuracy as harmonics increase. In contrast, for $\sum_n^N I(n\omega)$, the SEP reduction is around 24%, suggesting that increasing harmonics has a comparatively smaller effect in this case.

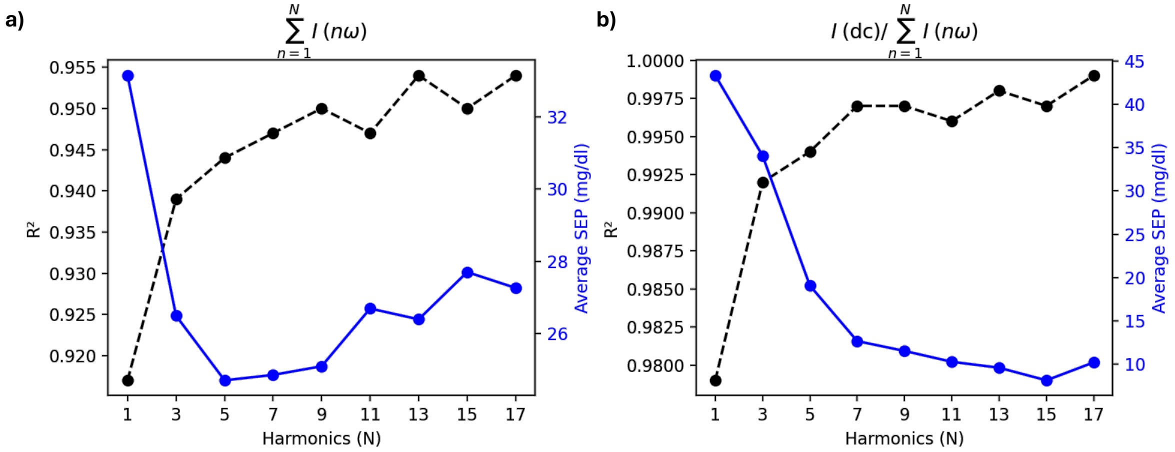

Figure S4. R^2 and average SEP of the non-linear regression for (a) $\sum_n^N I(n\omega)$ and b) $I_{DC}/\sum_n^N I(n\omega)$ for harmonics up to $N = 17$.

S4 Investigation of different error sources

To evaluate the stability and reproducibility of the measurement system, a series of experiments and calculations was performed to identify potential sources of error, including variations in signal intensity arising from beamline fluctuations, the presence of a cuvette, and temperature dependence.

Intensity variations were analysed over 15 consecutive measurements under three conditions: a) the setup without a cuvette, b) the setup with an empty cuvette, and c) the setup with a cuvette filled with PBS. Each measurement was colour-coded from yellow (first measurement) to purple (last measurement) to facilitate visualization of temporal trends. The results, presented in Figure S5, provided insights into systematic variations introduced by the optical path and cuvette materials.

The standard deviation of intensity measurements was computed for each condition, yielding relative standard deviation values of 0.144% for the setup without a cuvette, 0.99% for the empty cuvette, and 0.148% for the PBS-filled cuvette. Laser fluctuations over time as well as temperature-induced changes in optical elements during measurement, were identified as the primary sources of errors. This conclusion was supported by the observation of intensity fluctuations in the absence of a cuvette, thereby ruling out birefringence alterations caused by the cuvette itself. The highest variability was observed when a cuvette filled with PBS was employed, with an average standard deviation of 0.14%, suggesting that additional contributions from cuvette birefringence and temperature-induced variations in PBS were present.

Temperature dependence was further investigated through theoretical calculations[2] to assess its impact on polarization rotation. The results, shown in Figure S6, indicated that small but consistent shifts in polarization rotation occur with increasing temperature.

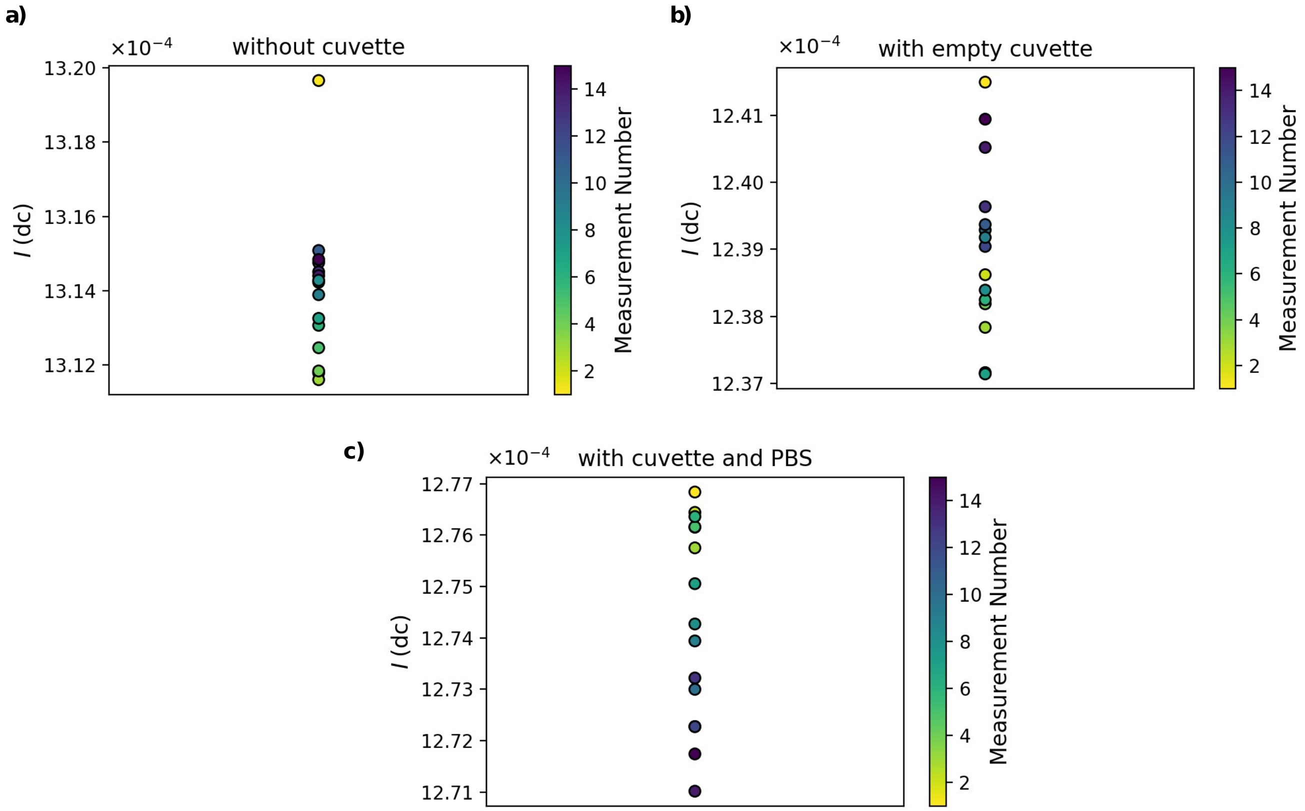

Figure S5. To visualize the effects of measurement variations, each data point is colour-coded from yellow to purple, representing the progression of 15 consecutive measurements. (a) Signal measured without a cuvette, (b) with an empty cuvette, and (c) with a cuvette filled with PBS. The first measurement is represented in yellow, and the last measurement in purple, showing the evolution of intensity variations over repeated measurements.

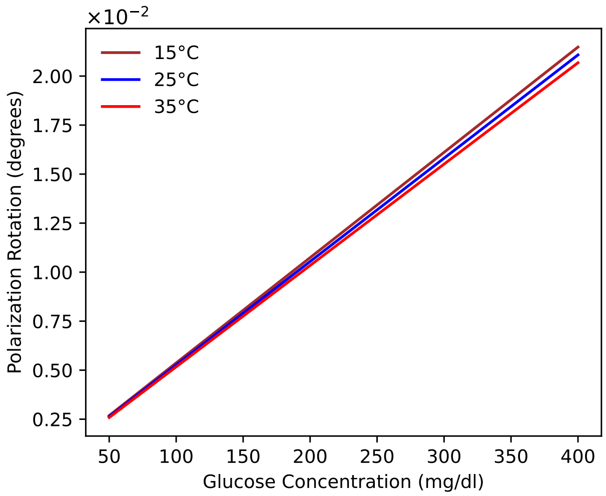

Figure S6. Calculated temperature dependence of the polarization rotation.

S5 MARD calculations

For a comparison with existing CGMs in addition to the SEP, the MARD was calculated as the mean of the absolute relative differences between the measured glucose concentration and the reference glucose concentration, using the formula:

$$\text{MARD} = \frac{1}{N}\sum_{i=1}^{N}\left|\frac{\text{Measured}_i - \text{Reference}_i}{\text{Reference}_i}\right| \times 100\% \tag{S1}$$

where N is the number of glucose concentration measurements. The MARD values as a function of glucose concentration are provided in Table S2.

Table S2. Measured glucose concentrations (c_G) and corresponding MARD values.

c_G (mg/dl)	MARD (%)
49.03	13.339
72.16	33.169
94.44	15.311
136.60	4.089
194.49	1.611
229.91	1.439
278.90	0.449
351.08	0.062
401.71	0.009

S6 Optimisation of the polarisation modulation angle θ_M

The effect of θ_M on the glucose concentration dependence of the signal can be analyzed using the simpler case of continuous polarisation modulation. For continuous sinusoidal polarisation modulation, such as that achieved with a Faraday modulator, the electric field $E(t)$ is expressed as follows[2,3]:

$$E(t) = -[\sin(\theta_M \cdot \sin(\omega t) + \phi)] \cdot E_0 \tag{S2}$$

where θ_M and ω are the modulation depth and frequency, respectively. E_0 represents the electric field amplitude of the light source. The phase shift introduced by the sample is represented by ϕ. Since both θ_M and ϕ are small, the approximation $\sin(x) \approx x$ applies:

$$E(t) \approx -[\theta_{\mathrm{M}} \cdot \sin(\omega t) + \phi] \cdot E_0. \tag{S3}$$

Using trigonometric identities, the output intensity can therefore be expressed as[2]:

$$I(t) \propto E^2(t) \approx \left[\underbrace{\left(\frac{\theta_{\mathrm{M}}^2}{2} + \phi^2 \right)}_{\mathrm{dc}} + \underbrace{2 \cdot \theta_{\mathrm{M}} \cdot \phi \cdot \sin(\omega t)}_{\omega} - \underbrace{\frac{\theta_{\mathrm{M}}^2}{2} \cdot \cos(2\omega t)}_{2\omega} \right] \cdot E_0^2 \cdot T \tag{S4}$$

The FFT decomposes the signal into its frequency components and extracts their amplitudes. In this process, only the prefactors of the sinusoidal terms remain, while the time-dependent functions are removed. Therefore, the ratio of DC and the odd frequency components will be:

$$\frac{I_{DC}}{I_\omega} = \left[\left(\frac{\theta_{\mathrm{M}}^2}{2} + \phi^2 \right) / 2 \cdot \theta_{\mathrm{M}} \cdot \phi \right] = \frac{1}{2\theta_{\mathrm{M}}} \phi + \frac{\theta_{\mathrm{M}}}{4} \frac{1}{\phi} \tag{S5}$$

From equation (7) of the main document, it is understood that ϕ represents glucose concentration c_G in a linear fashion. Therefore, equation (S5) can be rewritten in the form of equation (8) in the main document, with d and e defined as $1/2\theta_{\mathrm{M}}$ and $\theta_{\mathrm{M}}/4$, respectively. For the case of square-wave polarisation modulation, certain prefactors are altered; however, the dependence on θ_{M} remains unchanged.

Based on equation (S4) and subsequently equation (6), the ratio $\frac{d}{e}$ is found to be proportional to $\frac{1}{\theta_{m_i}^2}$, indicating that an increase in θ_m results in a decrease in $\frac{d}{e}$. In Figure S7, the experimentally obtained θ_m from the $\frac{d}{e}$ ratio in Figure 4 is assumed as the initial value, denoted as θ_{m_i}, which is experimentally determined to be below 1°. Subsequently, θ_{m_i} was systematically varied. As shown in Figure S7, four cases were plotted: $10\theta_{m_i}, 3\theta_{m_i}, \theta_{m_i}, \theta_{m_i}/10$.

A higher θ_{m_i} results in the appearance of a local maximum in the quadratic fit, where a single $I_{\mathrm{dc}}/\sum I_{\mathrm{odd}}$ value corresponds to two possible glucose concentrations. In contrast, relatively lower values of θ_m produce a more linear behaviour in the fitted curve, thereby improving the accuracy of glucose estimation. While decreasing θ_{m_i} enhances sensitivity—since glucose-induced rotation leads to more pronounced changes, achieving such small angles experimentally remains a challenge.

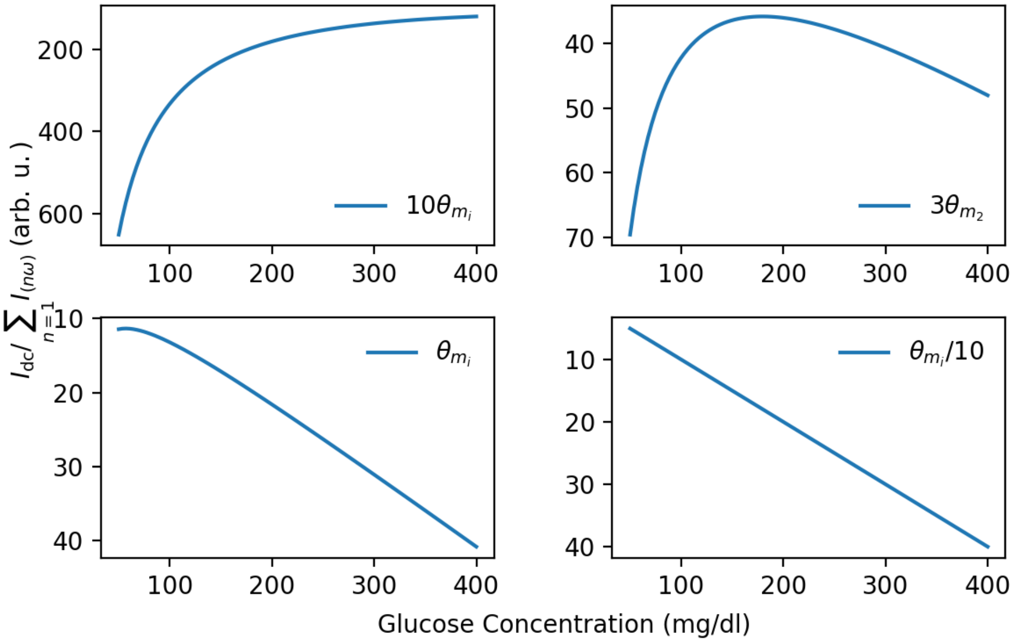

Figure S7. Effect of varying θ_{m_i} on the fitted curve of $I_{\mathrm{dc}}/\sum I_{\mathrm{odd}}$ as a function of glucose concentration. The cases plotted correspond to $10\theta_{m_i}$, $3\theta_{m_i}$, θ_{m_i}, and $\theta_{m_i}/10$. As θ_{m_i} increases, the fitted curve transitions from a quadratic shape, where a local maximum appears, to a more linear trend.

S7 Effect of beam size at the chopper

Figure S8 shows a sample of the waveform where for the left arm the beam was focused on the chopper and the right arm beam was collimated diameter to approximately 3.5 mm. With the unfocused beam, the gradual cutting of the chopper blade manifests via a smooth increase and a decrease of the intensity which is not equivalent to a smooth change of the polarization as in analogue modulation. The second parameter that leads to a similar result is the amplification of the photodiode which affects the rise- and fall time of the detector. Here, three different gain levels for the photodiode and their effect on the signal are shown. Therefore, the use of appropriate amplification is required for the photodiode detector.

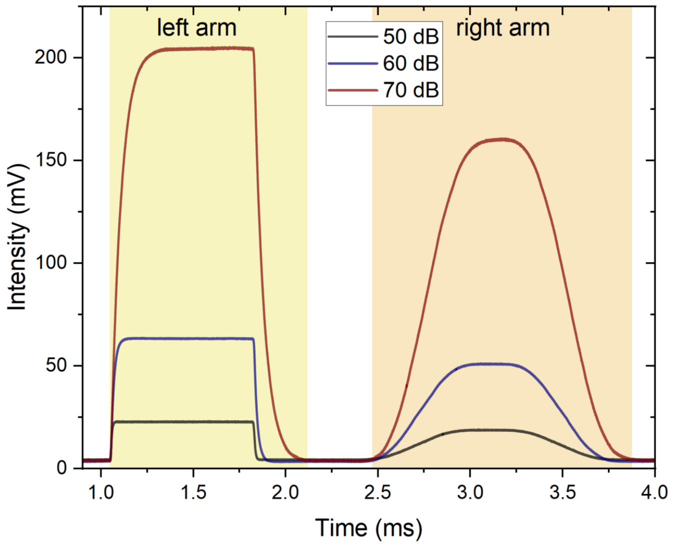

Figure S8. Sample waveform recorded for signal line with the beam in the left arm focused on the chopper and the right arm unfocused. Three different photodiode amplification gains of 50, 60, and 70 dB are used and their effect on the rise- and fall time of the detector is shown.

References

1. Thorlabs, Inc. Beam Splitter Cube, AR-Coated, 400-700 nm (BS010). https://www.thorlabs.de/thorproduct.cfm?partnumber=BS010. Accessed: 10 February 2024.

2. Stark, C., Behroozian, R., Redmer, B., Fiedler, F. & Müller, S. Real-time compensation method for robust polarimetric determination of glucose in turbid media. *Biomed. Opt. Express* **10**, 308, DOI: 10.1364/BOE.10.000308 (2019). Publisher: Optical Society of America.

3. Cameron, B. & Cote, G. Noninvasive glucose sensing utilizing a digital closed-loop polarimetric approach. *IEEE Transactions on Biomed. Eng.* **44**, 1221–1227, DOI: 10.1109/10.649993 (1997).
